# Study of Demographic Profile, Etiology, and Clinical Outcome in Patients Admitted With Acute Encephalitis Syndrome From the Western Part of India

**DOI:** 10.7759/cureus.23085

**Published:** 2022-03-11

**Authors:** Dhara B Roy, Harsh V Khatri

**Affiliations:** 1 Medicine, Smt. Nathiba Hargovandas Lakhmichand Municipal Medical College (NHLMMC), Ahmedabad, IND

**Keywords:** postinfectious adem, viral meningoencephalitis, autoimmune encephalitis, viral encephalitis, acute encephalitis syndrome

## Abstract

Background

Determining the etiology of encephalitis always remains a challenge to clinicians, and also, variables that predict outcome in acute phase settings are not known precisely. The autoimmune causes of acute encephalitis are increasing due to the availability of newer diagnostic markers, whereas earlier studies were primarily focused on infectious causes. We conducted a prospective study to determine the demographic profile, etiological aspect, and in-hospital outcome of patients admitted with acute encephalitis syndrome (AES) in our tertiary care center.

Materials and method

This observational prospective study was carried out at a tertiary care hospital between November 2016 and October 2018. With a sample size of 72, appropriate statistical analysis was done.

Results

The incidence of AES usually escalates during the rainy season, with arboviral etiologies being predominant. The majority of the patients with AES with a likely infectious etiology could not be diagnosed with presently available viral marker studies. Among various clinical variables, a low Glasgow Coma Scale (GCS) score on admission, a high CSF protein value, and diffusion restriction on brain MRI was associated with poor outcome.

Conclusion

Acute encephalitis and encephalitis-related mortality impose a considerable burden on current medical practice. The reported demographics of hospitalized patients with encephalitis may be changing, which are important factors to consider for etiological workup.

## Introduction

Acute encephalitis is a challenging syndrome worldwide to diagnose and manage given the heterogeneity of clinical presentations and the myriad of causative agents. Etiology varies from a wide range of viruses, fungus, bacteria, parasites, spirochetes, and chemical and toxin exposure. Over the past decade, numerous advances have uncovered novel infectious and autoimmune etiologies of encephalitis [1]. Despite such advances, in large studies, more than 50% of encephalitis cases typically remain without an identified etiology, posing additional challenges in delivering prognosis and treatment [1].

Encephalopathy refers to a clinical state of altered mental status, manifesting as confusion, disorientation, behavioral changes, or other cognitive impairments, with or without inflammation of the brain tissue [1]. Encephalopathy without inflammation can be triggered by several metabolic or toxic conditions [1]. In contrast, encephalitis is characterized by brain inflammation as a consequence of direct infection of the brain parenchyma [2], a postinfectious process (e.g., ADEM), or a noninfectious condition such as autoimmune encephalitis [1]. Acute onset fever, headache with or without focal neurological deficit, and CSF leukocytosis are in favor of acute encephalitis. In contrast, steady deterioration in sensorium without fever and the absence of CSF leukocytosis is a common presentation of encephalopathy [3].

The etiologies of acute encephalitis syndrome (AES) are divided into three broad groups: infective, post-infective (ADEM), and autoimmune. Viruses are the infectious agents most commonly associated with encephalitis. Herpes simplex virus (HSV1 and HSV2), other herpesviruses (human herpes virus-6 (HHV-6), Epstein-Barr virus (EBV), varicella-zoster virus (VZV), and cytomegalovirus), and adenovirus are common etiological DNA viruses. Among RNA viruses, influenza A, enterovirus, rabies, arbovirus (dengue, chikungunya, and Japanese encephalitis (JE)), and retrovirus (HIV) infections are common in Indian adults. Some bacterial infections, such as *Mycobacterium tuberculosis*, *Mycoplasma pneumoniae*, *Listeria monocytogenes*, *Leptospira*, and *Salmonella typhi*, can also present as AES. Rickettsial, fungal, and parasitic infections should also be considered in differential diagnosis [3]. Postinfectious or postimmunization ADEM has been seen with coronavirus, coxsackievirus, Epstein-Barr virus, herpes simplex virus, cytomegalovirus, human herpes virus-6, hepatitis A, H influenza A/B, parainfluenza, measles, rubella, West Nile virus, *Rotavirus*, *Mycoplasma pneumoniae*, *Bartonella henselae*, *Chlamydia*, *Leptospira*, and *Rickettsia* [3]. Autoimmune causes can have antibodies against AMPA receptor, NMDA receptor, gamma-aminobutyric acid B receptor, voltage-gated potassium channel complex, and LGi1 [4].

In India, the causative agent for AES varies with season and geographical location and affects the younger population. In recent times, AES cases in India have shifted toward JE etiology [5]. Toxin-mediated illness due to toxin in litchi fruit has also been hypothesized. In the United States, in the last decade, the West Nile virus has emerged as an important viral cause of encephalitis [6].

Obtaining a comprehensive case history, including recent and remote travel, animal contacts, and insect exposure, and careful analysis of presenting symptoms, signs, and laboratory and neuroimaging findings are crucial before performing additional expensive testing in a resource-limited country like India. CSF study data are also limited in analyzing CSF proteins with outcomes as shown by Roos [7]. In this study, we mainly focused on demographic profile, detailed clinical history, and thorough clinical examination. Combining them with CSF cytology, viral/autoimmune panel, and MRI, we tried to reach the diagnosis. In-hospital outcomes in terms of morbidity and mortality were compared in relation to etiology and neurological involvement on admission.

## Materials and methods

This prospective observational study included patients aged >15 years admitted at Sheth VS General Hospital from November 2016 to October 2018, if they met the inclusion criteria after informed consent was obtained. A total of 72 cases were studied (Table 1).

**Table 1:** Inclusion and exclusion criteria

Inclusion criteria	Exclusion criteria
Major criterion (required): Patients presented with altered mental status (defined as decreased or altered level of consciousness, lethargy, or behavioral change) lasting more than or equal to 24 hours with no alternative cause identified	Encephalopathy secondary to other causes, such as toxins, sepsis, or metabolic disorders
Minor criteria (two required for possible encephalitis; three required for probable or confirmed encephalitis)	Cases of bacterial or fungal meningitis with secondary encephalitic features (meningoencephalitis), patients with a final diagnosis of tuberculous meningitis as well as meningoencephalitis, and any patients who were diagnosed with encephalitis but subsequently were found to have an alternative confirmed diagnosis mimicking encephalitis, such as brain tumors
Minor criteria 1: Documented fever more than or equal to 38°C (100.4°F) within the 72 hours before or after presentation of generalized or partial seizures not fully attributable to a preexisting seizure disorder	HIV-positive patients
Minor criteria 2: New onset of focal neurological findings	
Minor criteria 3: Abnormality of brain parenchyma on neuroimaging suggestive of encephalitis that is either new from prior studies or appears acute in onset	
Minor criteria 4: Abnormality on EEG that is consistent with encephalitis and not attributable to another cause	

On admission, the Glasgow Coma Scale (GCS) was used to categorize patients into two categories. Those with a GCS score < 8 and those with a GCS score > 8. Further investigations included CSF examination, i.e., biochemical and cytological analysis and CSF for virological analysis, brain MRI, blood investigations such as complete blood count, renal function tests, and liver function tests with serological investigation for virus and bacteria. Specific investigations such as FilmArray (PCR) for the diagnosis of specific infective etiologies were done on CSF whenever patients did not have a financial constraint. We used one CSF aliquot (1.0 mL) to perform nucleic acid extraction with Qiagen DNA and RNA extraction kits (Qiagen, Hilden, Germany) using standard techniques. We performed RT-PCR on extracted RNA for enteroviruses and extracted DNA for herpesviruses. We performed conventional PCR for flaviviruses. We used a second aliquot of the CSF sample (1.0 mL) to test for IgM antibodies against Japanese encephalitis virus, dengue virus, West Nile virus, and varicella-zoster virus using commercial IgM capture ELISA kits. The second line tests for measles and mumps were performed with an IgM ELISA on CSF samples using commercial kits. If the CSF RT-PCR was positive for an etiological agent, it was considered as diagnostic. The CSF IgM ELISA results were interpreted for PCR-negative cases, and serum IgM ELISA tests were considered as diagnostic only if all CSF-based test results were negative. Those with inconclusive results were categorized as unknown etiologies. Serum investigations for antibody detection of common arboviral diseases such as dengue and chikungunya were done whenever suspicion remained high. A corneal impression test was performed in patients with possibility of rabies encephalitis. Other appropriate investigations were done as per the patient’s clinical presentation.

Patient treatments were monitored, and in-hospital outcomes at the time of discharge were recorded in terms of the modified Rankin Scale (MRS), with a scale of less than 3 classified as good outcome and a scale of equal or more than 3 as poor outcome. Patient outcomes were compared with different prognostic variables, such as underlying etiology, seasonal variation, type of convulsion, GCS score on admission, a requirement of mechanical ventilation, mean CSF protein value, and MRI findings.

Statistical methods

Descriptive and inferential statistical analyses have been carried out in the present study. The results of continuous measurements are presented as mean USD (minimum-maximum), and the results of categorical measurements are presented as numbers (%).

Statistical software SAS 9.2 (SAS Institute Inc., Cary, NC, USA), SPSS 15.0 (SPSS Inc., Chicago, IL, USA), Stata 10.1 (StataCorp LLC, College Station, Texas, USA), MedCalc 9.0.1 (MedCalc Software Ltd., Ostend, Belgium), Systat 12.0, and R Environment version 2.11.1 were used for the analysis of data, and Microsoft Word and Excel (Microsoft Corp., Redmond, WA, USA) have been used to generate graphs, tables, etc. Pearson correlation, chi-square/Fisher’s exact test, and analysis of variance (ANOVA) were done. Ethical approval was taken from the institutional ethics committee. Written consent was taken from each study subject. For those who were illiterate, thumb impressions were taken in front of a witness. All information collected was kept confidential.

For clinical signs and symptoms, sex distribution, etiology, types of convulsions, and seasonal distribution proportions were calculated. Mean values were calculated for age, CSF protein, CSF glucose, CSF white blood cells, and preceding illness duration.

To see an association of prognostic variables such as convulsions, GCS scores, and a requirement of mechanical ventilation with outcomes, statistical tests and chi-square tests were applied as and when required. The outcomes of the patients in the study, MRI findings, and related outcomes, as well as complications associated with AES, were calculated in proportion.

## Results

Clinical profile and presentation

The highest number of cases were recorded in the rainy season. Arboviral etiologies such as dengue remained common. The mean age of the patients that took part in the study was 35.9 (SD: 18.89) years. Forty-four (61%) patients were less than 40 years of age. The mean age in males was 36.3 years (SD: 18.99), while that in females was 35.6 years (SD: 18.44). Out of the 72 patients, 46 were male and 26 were female (Table 2).

**Table 2:** Age-wise distribution of patients with AES

Age	n (%)
0–20	21 (29.16)
21–40	23 (31.94)
41–60	19 (26.38)
>60	9 (12.5)
Total	72

Preceding illness or event was found in a total of 14 (19.44%) patients. Upper respiratory tract infection (URTI) and acute gastroenteritis (AGE) were found in three (4.16%) and four (5.55%) patients. Two (2.77%) patients were diagnosed with rabies encephalitis, and it was associated with a positive history of a dog bite in the past. The average duration of the preceding illness was 6.1 days.

A large majority of patients had a fever (97.2%) and altered sensorium (97.2%) at the presentation in our study, followed by convulsion (74%), headache (68%), and vomiting (58%). Myalgia and rash seen commonly with viral infections were found in six (8%) and five (7%) patients, respectively. Neurological deficits in terms of hemiparesis or paraparesis were found in 10 patients.

In 46 (63.88%) patients, patient etiology remained unspecified. Dengue viral infection was the most common etiology found among known etiology cases, followed by autoimmune NMDA encephalitis, herpes simplex, and rabies encephalitis. Two patients were found to have mixed etiology for acute encephalitis, both of them having autoimmune etiology and dengue infection (Table 3).

**Table 3:** Demographic profile of AES

Demographic variables	n (%)
Age	35.9 ± 18.89 (mean ± SD)
Male sex	46 (63.88)
Preceding illness	
Upper respiratory tract infection (URTI)	3 (4.16)
Acute gastroenteritis (AGE)	4 (5.55)
Herpetic lesions	4 (5.55)
Chickenpox	2 (2.77)
Epididymo-orchitis	1 (1.38)
Chikungunya	1 (1.38)
Etiology	
Autoimmune	7 (9.72)
Herpes simplex virus 1 (HSV1)	3 (4.16)
Varicella-zoster virus (VZV)	1 (1.38)
Rabies	2 (2.77)
Chikungunya	1 (1.38)
Dengue	13 (18.05)
Japanese encephalitis (JE)	2 (2.77)
Unspecified	46 (63.88)
On admission	
Glasgow Coma Scale (GCS)	9.33 ± 3.95 (mean ± SD)
Leucocytosis	37 (51.37)
Thrombocytopenia	12 (16.66)
Clinical profile	
Fever	70 (97.22)
Headache	36 (50)
Vomiting	29 (40.27)
Photophobia	8 (11.11)
Abnormal behavior	15 (20.83)
Altered sensorium	70 (97.22)
Rash	5 (6.94)
Limb weakness	9 (12.5)
Mechanical ventilation	38 (52.77)

Seizures were categorized into generalized and focal seizures. Status epilepticus was included in the above two groups based on their subtype was found in 14 patients. We had one patient with nonconvulsive status epilepticus (NCSE) (Table 4).

**Table 4:** Types of seizures in AES

Seizure	n (%)
Focal	13 (18.05)
Generalized	39 (54.16)
Nonconvulsive	1 (1.38)
Total	53 (73.61)

Hospital course and outcome

We divided the patients into two major groups based on their Glasgow Coma Scale (GCS) score at admission, and later on, outcomes were determined based on their neurological function at discharge or LAMA, as well as after one month of the hospital stay by the modified ranking score.

Based on the GCS, two groups were made; one of them had patients with a GCS score < 8 and the other with a GCS score > 8. A good outcome is defined as MRS < 2 at a particular point of time (at the time of discharge/discharge against medical advice (DAMA) from a hospital or one month post-discharge/DAMA). A bad outcome is defined as MRS > 2 at a particular point of time. Complete recovery means modified Rankin Scale (MRS) of less than or equal to 2 after one month of discharge or DAMA. Recovery with deficit includes MRS 3-5 and/or cognitive impairment.

Thirty-eight (52.77%) patients required mechanical ventilation. Out of the 72 patients, 46 (64%) patients were discharged in a hemodynamically stable condition, seven (10%) patients took discharge against medical advice, and 19 (26%) patients expired during the hospital stay. Out of 45 patients, 39 (54%)patients had complete recovery (MRS less or equal to 2) one month post-discharge, and seven (10%) recovered with a deficit (MRS 3 to 5). A total of 45 (62.5%) patients had a good outcome at one month post-discharge, and a total of 27 (37.5%) patients had a poor outcome.

The mean protein in CSF in our study was 78.16 (SE: 8.12) mg/dL (three patients had a CSF protein of 280 mg/dL and higher, which were not taken into account). The mean sugar in CSF was 82.4 mg/dL (SE: 4.5 mmol/L), whereas the mean white blood cells in CSF in our studies was 37.55 mm^3^. Only four patients were found with CSF glucose < 40 mg/dL. Out of these four, one patient had ADEM, two had AE unidentified, and one had NMDA.

MRI changes

The brain MRI findings in the patients were divided based on the area of involvement into six categories and one category for normal MRI findings. The outcome was compared for each area of involvement (Table 5).

**Table 5:** Area distribution on MRI in relation to patient outcomes

Site	Good	Poor	Total
Cortical	7	16	23 (31.94%)
Subcortical	12	7	19 (26.38%)
Brain stem	5	3	8 (11.11%)
Cerebellum	1	1	2 (2.77%)
Basal ganglia	3	5	8 (11.11%)
Thalamus	2	1	3 (4.16%)
Normal	24	5	29 (40.27%)

Normal MRI was the most common brain MRI finding in our study. For those with an abnormal brain MRI study, the most common area of involvement was cortical, followed by subcortical, basal ganglia, and brain stem.

## Discussion

The AES spectrum includes patients of all age groups and both sexes. In our study, the mean age was 35.95 (SD: 18.89) years. Hence, a relatively younger age population was involved in our study. On the contrary, a similar paper by Singh et al. carried out at the Mayo Clinic from January 2000 to December 2012 had a median age of 58 (range: 41.8-69) years [8]. The demonstrated mean age from another similar study by Joshi et al. conducted in rural central India was found to be 40.2 (SD: 18.3) years [9].

The younger age at our setup may be attributed to underlying etiologies that the patients with AES had. Season trends, as well as preceding illnesses, contributed the maximum for the provisional diagnosis, especially in a resource-limiting setup like ours. During the rainy season and around that period, viral causes such as flaviviruses and chikungunya remained prominent.

In 36% of the patients, etiologies were confirmed. This is much lower when compared to 55% obtained by the National Institute of Mental Health and Neurosciences (NIMHANS) AES Surveillance Network 2014-2018, which had a sample size of 6870 [10]. Additionally, in the paper by Singh et al., causes were discovered in 70.2% of the patients (n = 198) [8].

Infective etiology was found in 22 (30.55%) patients. On the other end, autoimmune and unspecified causes were responsible for four (5.55%) and 46 (63.88%), respectively, in our study. Two patients were found to have mixed etiology for acute encephalitis, both of them having autoimmune etiology and dengue infection. Rare etiologies such as Nipah, Chandipura, Zika, Influenzae, and Crimean-Congo hemorrhagic fever virus were absent probably due to the epidemiological determinants of the above infection and the small sample size of our study (n = 72) compared to the NIMHANS AES Surveillance Network (n = 6870) (Table 6) [10].

**Table 6:** GCS and outcome correlation

	Good outcome	Poor outcome	Total	Chi-square test = 8.72 (p < 0.05)
GCS < 8	11 (41%)	16 (59%)	27 (100%)	
GCS > 8	34 (75%)	11 (25%)	45 (100%)	
			72	

Patients with low GCS scores on admission (<8) were associated with poor outcomes, and this was statistically significant (chi-square test = 8.72). As mentioned previously, the study by Singh et al. also showed that a GCS score of less than or equal to 8 is associated with poor outcome (odds ratio = 5.06, p < 0.05) (Table 7) [8].

**Table 7:** Clinical significance of convulsion in patients with AES

	Good	Poor	Total	Chi-square test = 2.77 (p > 0.05)
Convulsion total	33	18	51	
Status epilepticus	7	11	18	

Although more proportion of patients with status epilepticus had poor outcomes than total convulsions, the outcome of patients based on convulsion in general and status epilepticus was found to be statistically insignificant.

In a study by Misra et al., out of the 210 patients, 90 (43%) had convulsions and 62 (30%) suffered from status epilepticus. Thus, it can be concluded that seizures were more common in our study when compared to the study by Misra et al., where status epilepticus was frequent [11].

When patients were divided into two groups based on their CSF protein values (in mg/dL), it was found that CSF protein values > 60 mg/dL were associated with good outcomes (67%) compared to CSF protein values < 60 mg/dL (60%) (Table 8).

**Table 8:** Relationship of CSF protein with outcomes

	CSF protein > 60 mg/dL (N = 30)	CSF protein < 60 mg/dL (N = 40)
Poor outcome	12 (40%)	13 (33%)
Good outcome	18 (60%)	27 (67%)

The brain MRI findings in the patients were divided based on the area of involvement into six categories and one category for normal MRI findings.

When brain MRI findings were compared with the outcomes, those patients with a normal brain MRI were more likely to have good outcomes (83%) than patients with abnormal imaging.

Cortical involvement was found in 23 patients, of which 16 (69%) of them have a poor outcome. From a total of 19 patients with subcortical involvement, seven (37%) patients had a poor outcome. Out of eight patients with basal ganglia involvement, five (62%) had a poor outcome. Of eight patients with brain stem involvement, three (37%) had a poor outcome. Three patients had thalamic involvement, and one had a poor outcome. Out of the two patients with cerebellar involvement, only one had a poor outcome.

In a study by Singh et al., which included 198 patients with acute encephalitis, out of 71 patients with cortical involvement, 32 (45%) had a poor outcome, and out of 88 patients with FLAIR/T2 abnormalities, 47 (53%) had a poor outcome [8].

Patients’ requirement of mechanical ventilation during the hospital stay and their association with the outcomes (good or poor) based on MRS were compared (Table 9).

**Table 9:** Relationship of the need for ventilator support with outcomes

	Good	Poor	Total	Chi-square test = 22.6 (p < 0.05)
Ventilator	14 (37%)	24 (63%)	38	
No ventilator	31 (91%)	3 (9%)	34	
Total	45	27	72	

The chi-square test value (22.6) is more than the table value (3.84); hence, it suggests that the requirement of mechanical ventilation was associated with poor outcomes, and this observation is statistically significant.

In the study by Singh et al., of 50 patients who required mechanical ventilation and/or intubation, 40 (80%) patients had a poor outcome and 10 (20%) had a good outcome (p < 0.0001) [8].

Limitations of the study

In our study, only a few causative agents were screened for, with the help of the commercial kits. Many agents, whose incidence is questionable, were left out due to financial and resource limitations. There are still many cases of AES with less known or screened agents.

In the cases of mortality, necropsy or postmortem examination was not done, further limiting our chances to diagnose the responsible agents. With the development in science and future availability of resources, we can hope to understand the etiology of many of these patients.

## Conclusions

A relatively younger age group (mean age: 21-40 years) of patients of either gender were admitted with AES. Viral infectious diseases remain the most common etiology, followed by autoimmune. A maximum number of infectious cases, including dengue and chikungunya, were reported during the rainy season. The other infectious agents were HSV1, JE, VZV, and rabies, recorded sporadically. Arboviral diseases and rabies are very rare causes of encephalitis globally. Mortality was 26%, and the overall poor outcome was recorded at 36.5% one month post-discharge. The factors associated with poor outcomes were low GCS score (<8) on admission, high CSF protein count, abnormal MRI suggestive of focal cortical or subcortical area of involvement, and requirement of mechanical ventilation. In conclusion, AES is a considerable cause of morbidity and mortality involving young adults. Knowledge of the spectrum of etiology in a particular area, early diagnosis, and specific treatment will help in reducing morbidity and mortality.
